# Ultra‐Processed Food Dietary Patterns and Obesity: A Cross‐Sectional Analysis of NHANES 2021–2023

**DOI:** 10.1002/hsr2.73062

**Published:** 2026-08-19

**Authors:** Nadia Daniel, Carmen Piernas, Paul Sherliker, Carolina Abreu de Carvalho, Jennifer Carter

**Affiliations:** ^1^ Medical Sciences Division University of Oxford Oxford UK; ^2^ Department of Biochemistry and Molecular Biology II Faculty of Pharmacy, University of Granada Granada Spain; ^3^ Center for Biomedical Research, Institute of Nutrition and Food Technology University of Granada Granada Spain; ^4^ Instituto de Investigación Biosanitaria ibs.GRANADA Granada Spain; ^5^ CIBER Physiopathology of Obesity and Nutrition (CIBEROBN), Instituto de Salud Carlos III Madrid Spain; ^6^ Nuffield Department of Population Health University of Oxford Oxford UK; ^7^ Departamento de Saúde Pública Universidade Federal do Maranhão São Luís Brazil; ^8^ Postgraduate program in Public Health Federal University of Maranhão São Luís Brazil; ^9^ Health Data Research UK (HDRUK‐Oxford) University of Oxford Oxford UK

**Keywords:** body mass index, dietary patterns, NHANES, obesity, ultra‐processed food, waist circumference

## Abstract

**Background and Aims:**

Ultra‐processed foods (UPFs) are widely consumed and have been linked to obesity. It is unclear whether different UPF dietary patterns vary in their associations with adiposity. This study aimed to identify UPF dietary patterns and examine their associations with adiposity in a nationally representative sample of United States adults.

**Methods:**

In 4003 adults from NHANES 2021–2023, UPF dietary patterns were derived using k‐means clustering, and associations of total UPF intake and dietary patterns with BMI and waist circumference were assessed using adjusted survey‐weighted linear regression.

**Results:**

Each 5% increase in energy intake from UPFs was associated with a 0.25 kg/m^2^ higher BMI (95% CI: 0.16–0.34) and a 0.60 cm greater waist circumference (95% CI: 0.39–0.81). Three UPF dietary patterns were identified: “Fast Food,” “Treats and Diet Soda,” and “Lower UPF.” Compared with the “Lower UPF” pattern, both “Fast Food” (BMI + 3.20 kg/m^2^; waist circumference +7.76 cm) and “Treats and Diet Soda” (BMI + 2.83 kg/m^2^; waist circumference +6.78 cm) patterns were associated with higher adiposity. No significant differences were observed between the two high‐UPF patterns.

**Conclusion:**

Higher UPF consumption was associated with greater adiposity across UPF dietary patterns. These findings from cross‐sectional data suggest that overall UPF intake may be an important marker of adiposity, but longitudinal studies are needed to assess temporality and causality.

**Abbreviations**
BMI: body mass index
FNDDS: food and nutrient database for dietary studies
NHANES: national health and nutrition examination survey
PSU: primary sampling unit
REE: ratio of reported energy intake to estimated energy requirements
UPF: ultra‐processed foods

## 1 Introduction

The Nova classification categorizes foods by the extent of industrial processing into four groups: unprocessed or minimally processed foods, processed culinary ingredients, processed foods, and ultra‐processed foods (UPFs) [1]. UPFs are characterized as energy‐dense, high in unhealthy types of fat, refined starches, free sugars and salt, and low in protein, fiber, and micronutrients [1]. Since the 1980s, global UPF consumption has risen sharply, particularly in high‐income countries such as the United States, where UPFs contribute a substantial proportion of dietary energy [2, 3]. Similar trends are emerging in low‐ and middle‐income countries undergoing nutrition transition [2].

Accumulating research suggests that increased UPF consumption is linked with rising obesity rates [3]. Cross‐sectional studies and prospective cohort studies across diverse populations consistently find that higher UPF intake is associated with increased BMI, waist circumference, and body fat [4, 5, 6, 7, 8]. Further experimental evidence from randomized controlled trials demonstrates that UPF diets lead to higher energy intake and weight gain compared to matched minimally processed diets [9, 10, 11]. These associations often persist after adjustment for dietary mediators, suggesting that ultra‐processing has effects on adiposity beyond nutrient composition.

Although total UPF intake is consistently associated with obesity, it remains unclear whether all UPFs carry equal risk. Evidence from prospective studies on type 2 diabetes and cardiometabolic outcomes suggests heterogeneity across UPF subtypes. In the European Prospective Investigation into Cancer and Nutrition (EPIC) cohort, higher intake of savory snacks and animal‐based UPFs was associated with increased type 2 diabetes risk, whereas breads and breakfast cereals were associated with lower risk [12]. Similarly, analyzes from three large prospective United States cohort studies reported strong positive associations for sugar‐sweetened beverages, but null or weaker associations for UPF breads and cereals [13]. Dietary pattern analysis is well established in nutrition epidemiology [14, 15], but less work has examined whether patterns derived specifically from UPF food groups differ in their associations with obesity‐related outcomes.

To address this gap, we conducted a cross‐sectional analysis of a nationally representative sample of United States adults using data from NHANES 2021 to 2023 [16]. We aimed to derive dietary patterns based specifically on UPF groups and evaluate whether these UPF‐specific consumption profiles differed in their associations with BMI and waist circumference.

## 2 Methods

A cross‐sectional analysis was conducted of adults (≥ 18 year) participating in the 2021–2023 National Health and Nutrition Examination Survey (NHANES). NHANES employs a complex, multistage probability sampling design to obtain a nationally representative sample of the civilian, non‐institutionalized United States population. Detailed methods have been published elsewhere [17]. In the 2021–2023 cycle, the household screener response rate was 65.6%, with 34.5% completing the household interview and 25.6% completing the examination (“NHANES Response Rates and Population Totals,” n.d.). The National Center for Health Statistics Research Ethics Review Board approved the NHANES protocol, and all participants provided written informed consent.

For this analysis, we restricted to adults with complete dietary recall data and non‐missing outcome and covariate information.

This study is reported in accordance with the STROBE guidelines for observational studies.

### 2.1 Dietary Intake

Dietary intake was assessed using two 24‐h dietary recalls administered using the U.S. Department of Agriculture's Automated Multiple‐Pass Method (AMPM) [17]. For the 2021–2023 NHANES cycle, both recalls were conducted by telephone. The first recall was scheduled 3–7 days after the Mobile Examination Center visit, and the second 3–10 days later on a different day of the week.

Reported foods and beverages were coded using the Food and Nutrient Database for Dietary Studies (FNDDS) and linked to USDA Standard Reference ingredient codes (“FNDDS: USDA ARS,” n.d.). Composite dishes were disaggregated into ingredients, with gram weights proportionally recalculated to match the total reported food weight [18]. Nova classification was assigned at the ingredient level, rather than at the whole preparation or food level. For example, the food “Beans soup, NFS” was disaggregated into the ingredients “Beverages, water, tap, drinking,” “Beans, black, mature seeds, cooked, boiled, without salt,” “Mirepoix, cooked, as ingredient,” “Vegetable oil, NFS,” “Salt, table,” with each ingredient assigned to a Nova group. Mixed dishes were assumed to be homemade unless clearly identified as ready‐to‐eat. Frozen meals, Lunchables, and vending machine foods were classified at the whole preparation level. Nutrient and energy values were summed across Nova groups to generate participant‐level estimates of energy intake from each Nova category [18].

The primary exposure was UPF intake, calculated as the percentage of total energy intake from Nova Group 4 items (%kcal) averaged across the two dietary recalls. UPF intake was modeled as a continuous variable and as dietary patterns derived from foods or ingredients classified as UPF (described below).

### 2.2 UPF Dietary Patterns

Participants reporting consumption of at least one ultra‐processed food were included in a k‐means clustering algorithm to derive UPF dietary patterns. Reported foods were grouped into 33 food groups (Supporting Information Table S1); food groups consumed by less than 1% of the sample were excluded, representing 6% of all reported UPF items (Supporting Information Table S2). Food group variables were standardized by centering to a mean of 0 and scaling to a standard deviation of 1.

Candidate clustering solutions were evaluated by varying the number of clusters and comparing their statistical performance and interpretability. The elbow plot was used to assess reductions in total within‐cluster sum of squares across increasing values of k (Supporting Information Figure S1), and average silhouette width was inspected as a measure of cluster separation (Supporting Information Figure S2). K‐means models were fitted using 100 random initial configurations (nstart = 100) and a maximum of 1000 iterations, with the retained solution corresponding to the partition with the lowest within‐cluster sum of squares.

The elbow plot indicated a clear inflection at k = 3, whereas the silhouette criterion favored k = 2. The 2‐, 3‐, and 4‐cluster solutions were therefore compared in detail. The 2‐cluster solution was considered overly coarse because it primarily separated participants according to overall level of UPF consumption and merged distinct high‐UPF consumption profiles into a single cluster (Supporting Information Table S3). The 4‐cluster solution provided limited additional improvement in within‐cluster fit and appeared to over‐partition the data, generating a small and less interpretable cluster; notably, one cluster comprised only 4.8% of the sample (Supporting Information Table S4). The 3‐cluster solution was therefore retained as the best compromise between statistical support, parsimony, and nutritional interpretability (Supporting Information Table S5).

### 2.3 Outcomes

Anthropometric outcomes were assessed by trained health technicians in the Mobile Examination Center using standardized protocols [17]. Weight (kg) was measured in duplicate with a digital scale, and standing height (cm) with a stadiometer. Waist circumference (cm) was measured in duplicate at the uppermost lateral border of the iliac crest in the mid‐axillary line, using a steel measuring tape with the participant standing and arms folded across the chest. If the first two measurements differed beyond protocol‐specified limits, a third measurement was taken, and the mean of the valid measures was used. BMI (kg/m^2^) was calculated as weight (kg) divided by height squared (m^2^). Both BMI and waist circumference were analyzed as continuous variables.

### 2.4 Covariates

Socio‐demographic covariates included age and age^2^, sex, race/ethnicity, educational attainment, ratio of family income to poverty, current smoking status, alcohol intake, moderate‐to‐vigorous physical activity, and chronic disease status (Supporting Information Note S1). Dietary covariates included total energy intake, total sugars, saturated fat, polyunsaturated fat, and fiber. The ratio of reported energy intake to estimated energy requirements (REE) was calculated using Institute of Medicine equations [19].

### 2.5 Statistical Analysis

Sociodemographic, dietary, and behavioral characteristics were summarized overall and by quartiles of UPF intake. Quartiles were used for descriptive analyzes, while UPF intake was modeled as a continuous variable in regression analyzes. For interpretability, the effect of continuous UPF intake was expressed per 5% increase in total energy intake from UPF (%kcal).

Unweighted counts and survey‐weighted proportions were reported for categorical variables, and survey‐weighted means with standard errors for continuous variables. Differences across UPF quartiles and dietary patterns were assessed using Rao‐Scott adjusted chi‐squared tests for categorical variables and survey‐weighted linear regression for continuous variables.

To assess potential selection bias, two sensitivity analyzes were conducted. First, adults included in the final analytic sample were compared with adults excluded because of missing dietary, anthropometric, cluster, or covariate data. Second, adults with valid BMI and waist circumference measurements were compared with adults missing BMI and/or waist circumference. Comparisons included demographic, socioeconomic, behavioral, and health characteristics, with between‐group differences assessed using Rao‐Scott adjusted chi‐squared tests for categorical variables and survey‐weighted linear regression for continuous variables.

Confounders were selected a priori based on biological plausibility and prior literature. Base models adjusted for age, age^2^, sex, and race/ethnicity. Extended models additionally adjusted for education, smoking status, and the REE. Dietary mediators (total sugars, saturated fat, and fiber) were included in separate models to assess attenuation along hypothesized pathways.

Weekend recall coverage was quantified, and as a sensitivity analysis, the continuous UPF models were additionally adjusted for the number of weekend recalls.

All tests were two‐sided with a significance threshold of α = 0.05. The complex, multistage survey design of NHANES was incorporated using strata, primary sampling units, and 2‐day dietary weights (“NHANES Survey Methods and Analytic Guidelines,” n.d.). Analyzes were performed in R (version 4.4.3) using the *survey* package (version 4.4‐2).

## 3 Results

The NHANES 2021–2023 dataset included 5878 participants. After excluding individuals younger than 18 years and restricting to those with complete dietary, anthropometric, and covariate data, the final analytic sample comprised 4003 adults (Supporting Information Figure S3). The mean age was 48.7 years (standard error 0.5), and 62% identified as non‐Hispanic White. 58.5% of adults had at least 1 weekend dietary recall. On average, 54.3% of total energy intake came from UPF, while mean BMI and waist circumference were 29.9 kg/m^2^ (standard error 0.3) and 101.2 cm (standard error 0.8), respectively (Table 1).

**Table 1:** Sociodemographic, lifestyle, dietary, and adiposity indicators of United States adults by quartiles of percentage of energy intake from ultra‐processed foods, NHANES 2021–2023. Mean (standard error) presented unless otherwise indicated.

Characteristic	Overall (*n* = 4003)	Quartile 1 (*n* = 1051)	Quartile 2 (*n* = 1078)	Quartile 3 (*n* = 984)	Quartile 4 (*n* = 890)	*p* value
Sex, *n* (%)						
Male	1761 (48.9)	461 (45.8)	477 (49.4)	443 (51.8)	380 (48.5)	0.20
Age (years)	48.7 (0.5)	49.9 (0.8)	50.7 (0.9)	48.0 (0.8)	46.3 (0.7)	<0.001
Race and ethnicity, *n* (%)						
Non‐Hispanic White	2517 (62.0)	559 (51.5)	691 (63.5)	656 (62.2)	611 (70.9)	
Non‐Hispanic Black	446 (11.1)	99 (9.0)	119 (11.6)	111 (12.0)	117 (12.0)	
Hispanic or other	1040 (26.9)	393 (39.5)	268 (24.9)	217 (25.9)	162 (17.1)	< 0.001
Education level, *n* (%)						
High school or below	1205 (32.3)	287 (27.6)	289 (28.0)	300 (33.9)	329 (39.6)	
Some college or associate degree	1210 (29.7)	270 (26.2)	304 (28.0)	315 (30.7)	321 (33.9)	
College graduate	1588 (38.1)	494 (46.3)	485 (44.0)	369 (39.6)	240 (26.5)	0.008
Income‐to‐poverty ratio	3.2 (0.1)	3.3 (0.1)	3.3 (0.1)	3.1 (0.1)	2.8 (0.1)	< 0.001
Moderate‐to‐vigorous physical activity (min/day)	51.4 (2.6)	51.9 (3.3)	47.5 (4.2)	48.9 (3.0)	57.4 (6.3)	0.36
Alcohol intake (drinks/day)	0.6 (0.03)	0.6 (0.1)	0.7 (0.1)	0.5 (0.0)	0.5 (0.1)	0.005
Smoking status, *n* (%)						
Current smoker	660 (17.6)	126 (13.6)	163 (15.2)	165 (19.0)	206 (23.1)	0.04
Chronic disease, *n* (%)						
Yes	2295 (48.2)	552 (43.0)	632 (49.3)	580 (48.4)	531 (51.9)	0.05
Dietary variables						
Ultra‐processed food (% total energy)	54.3 (0.6)	29.8 (0.4)	48.8 (0.2)	60.8 (0.2)	77.8 (0.3)	< 0.001
Total energy intake (kcal/day)	2058.6 (13.3)	1897.3 (34.3)	2047.5 (27.2)	2165.2 (27.4)	2119.9 (39.9)	< 0.001
Saturated fatty acids (g/day)	27.9 (0.3)	24.0 (0.6)	28.2 (0.6)	29.9 (0.5)	29.4 (0.6)	< 0.001
Polyunsaturated fatty acids (g/day)	20.2 (0.1)	17.8 (0.5)	20.1 (0.4)	21.6 (0.3)	21.4 (0.3)	< 0.001
Total sugars (g/day)	97.7 (1.3)	76.3 (2.2)	90.6 (1.3)	109.5 (3.4)	114.0 (3.7)	< 0.001
Sodium (mg/day)	3242.8 (25.4)	3036.9 (63.8)	3243.0 (50.8)	3402.0 (36.7)	3281.5 (66.6)	< 0.001
Dietary fiber (g/day)	16.2 (0.4)	18.8 (0.8)	16.8 (0.5)	15.7 (0.3)	13.5 (0.3)	< 0.001
Body mass index (kg/m^2^)	29.9 (0.3)	28.4 (0.5)	30.1 (0.4)	30.2 (0.3)	30.6 (0.4)	0.005
Waist circumference (cm)	101.2 (0.8)	97.6 (1.1)	102.0 (0.8)	102.0 (0.9)	103.1 (1.1)	0.002

Sensitivity analyzes indicated that excluded adults differed from the final analytic sample: they were younger, had lower income‐to‐poverty ratios, were less likely to be college graduates, reported higher moderate‐to‐vigorous physical activity, and had lower chronic disease prevalence (Supporting Information Table S6). Compared with adults with valid BMI and waist circumference measurements, adults missing BMI and/or waist circumference were older, had lower income‐to‐poverty ratios, reported lower physical activity, were less likely to be current smokers, and had higher chronic disease prevalence (Supporting Information Table S7).

Participants in the highest quartile of UPF intake tended to be younger, less educated, have a lower income‐to‐poverty ratio, consume fewer alcoholic drinks, smoke more, and obtain a higher proportion of energy from total sugars. Those in the lowest quartile had lower average values for total energy intake, sodium, and both saturated and polyunsaturated fatty acids (Table 1).

### 3.1 Cluster Analysis of UPF Dietary Patterns

Three clusters of UPF dietary patterns were derived (Supporting Information Table S5). Dietary Pattern 1, “Fast Food,” was characterized by increased consumption of fast foods, including pizzas, burgers, coated or breaded foods, fried potato dishes, and ultra‐processed fats. This dietary pattern also had a high intake of soft drinks, milk‐based drinks, snacks, dips and gravies, as well as cakes and biscuits. This dietary pattern accounted for 19.3% of the participants and had the highest consumption of UPF (66.0% of total energy intake).

Dietary Pattern 2, referred to as “Treats and Diet Soda,” represented 26.7% of the participants and was characterized by high intake of items such as bread, low‐calorie soft drinks, sugar substitutes, ice cream, desserts, chocolate, sweets, chips, crackers, ultra‐processed meat, spreads, ultra‐processed coffee‐ and tea‐based drinks, and breakfast cereal. This dietary pattern had a mean UPF contribution of 59.8% of total energy.

Dietary Pattern 3, “Lower UPF,” had the lowest UPF intake (46.1%) and comprised 54.0% of participants. This dietary pattern was characterized by a relatively higher consumption of whole‐grain bread and non‐dairy milk. The intake of discretionary foods, such as sausages, sandwiches, soft drinks, fruit drinks, fried foods, and sweets, was considerably lower than in the other two dietary patterns.

The dietary patterns with higher UPF intake, “Treats and Diet Soda” and “Fast Food,” included a greater proportion of males and younger individuals compared with the “Lower UPF” pattern. Participants in the “Fast Food” cluster were more likely to be non‐Hispanic Black, have lower education, report more physical activity, and have higher rates of smoking but fewer chronic diseases. In contrast, those in the “Treats and Diet Soda” cluster were typically older, more often college educated, and less likely to smoke, but still consumed a high proportion of UPF. Both high‐UPF patterns were characterized by higher mean intakes of total energy, sugars, and polyunsaturated fat, while the “Lower UPF” group had the lowest mean BMI and waist circumference as well as the lowest UPF contribution overall (Supporting Information Table S8).

### 3.2 Association of UPF Intake and Dietary Patterns With Obesity

**Figure 1:**
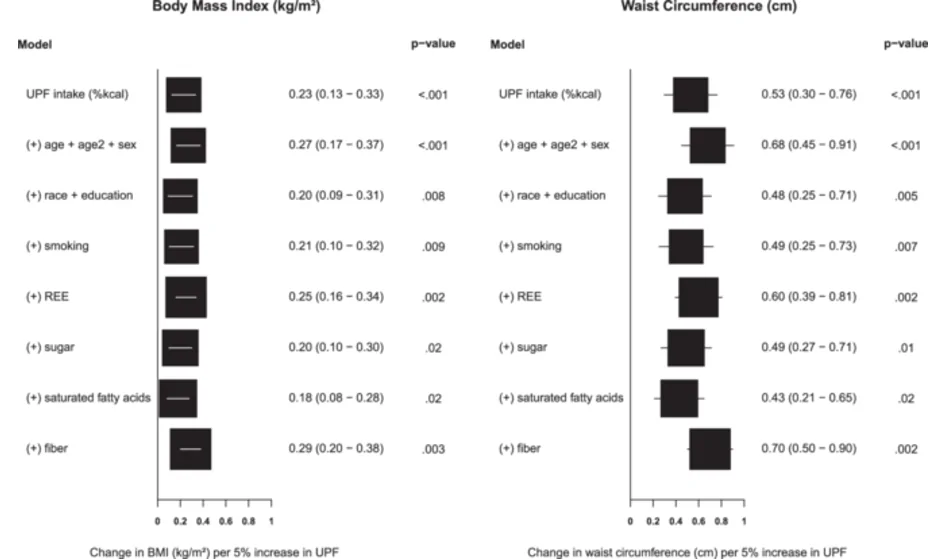
Ultra‐processed food intake and adiposity. Survey‐weighted associations of percentage of energy intake from ultra‐processed foods with body mass index (kg/m^2^) and waist circumference (cm) among United States adults (NHANES 2021–2023). Points represent regression coefficients per 5% increase in ultra‐processed food energy intake, with 95% confidence intervals.

The consumption of UPF (%kcal) was positively associated with BMI across all models. In the fully adjusted model, each 5% increase in UPF intake was associated with a 0.25 kg/m^2^ increase in BMI (95% CI: 0.16–0.34; *p* = 0.002). Further adjustment for intake of total sugars, saturated fatty acids, and fiber yielded comparable estimates. Consistent associations were observed for waist circumference, with a 0.60 cm increase (95% CI: 0.39–0.81; *p* = 0.002) per 5% increase in UPF consumption (Figure 1). Additional adjustment of the fully adjusted model for the number of weekend recalls did not materially alter these associations. Compared with the age‐ and sex‐adjusted model, additional adjustment for sociodemographic factors and smoking attenuated the association between UPF intake and BMI and waist circumference by 25.9% and 27.9%, respectively.

**Figure 2:**
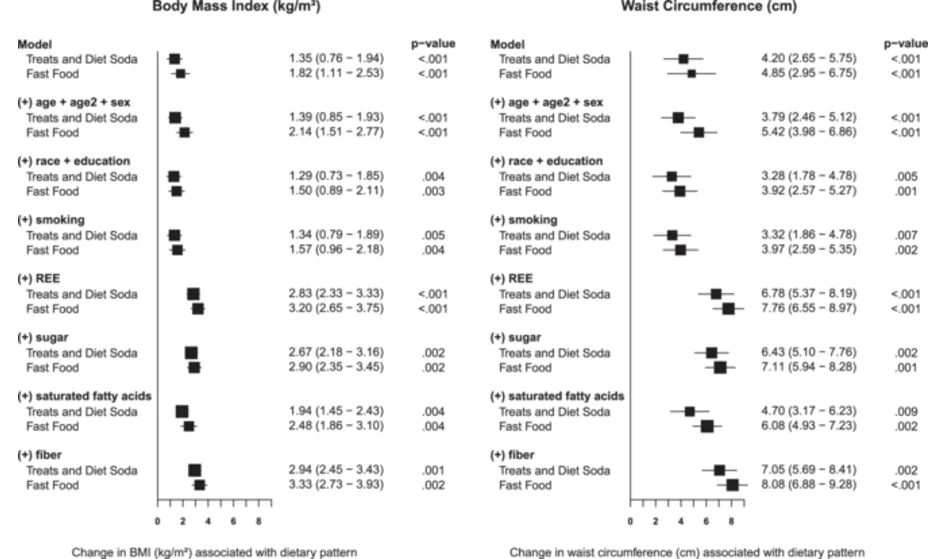
Adiposity by ultra‐processed food dietary pattern, using the lower UPF pattern as the reference. Survey‐weighted differences in body mass index (kg/m^2^) and waist circumference (cm) for the treats and diet soda and fast food patterns compared with the lower UPF pattern among United States adults (NHANES 2021–2023). Points represent regression coefficients with 95% confidence intervals.

The association between UPF dietary patterns and obesity indicators was assessed under two approaches. In the first, the “Lower UPF” pattern served as the reference. Belonging to the “Treats and Diet Soda” and “Fast Food” patterns was associated with 2.83 kg/m^2^ (95% CI: 2.33–3.33; *p* < 0.001) and 3.20 kg/m^2^ (95% CI: 2.65–3.75; *p* < 0.001) higher BMI, respectively, in fully adjusted models. Additional adjustment for total sugars, saturated fatty acids, and fiber produced similar estimates. For waist circumference, the fully adjusted associations were 6.78 cm (95% CI: 5.37–8.19; *p* < 0.001) in the “Treats and Diet Soda” pattern and 7.76 cm (95% CI: 6.55–8.97; *p* < 0.001) in the “Fast Food” pattern compared with the “Lower UPF” pattern (Figure 2).

**Figure 3:**
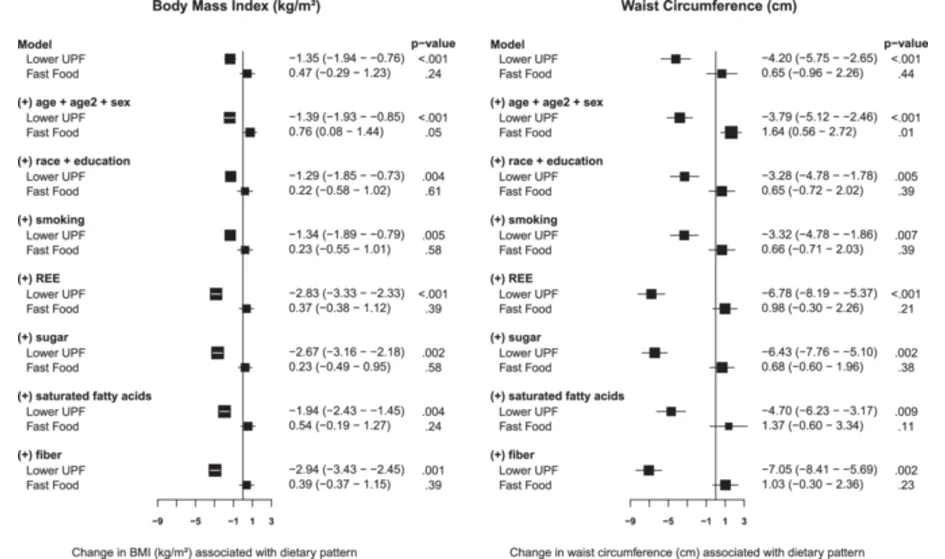
Comparison of ultra‐processed food dietary patterns, using the Treats and Diet Soda pattern as the reference. Survey‐weighted differences in body mass index (kg/m^2^) and waist circumference (cm) for the Fast Food and Lower UPF patterns compared with the Treats and Diet Soda pattern among United States adults (NHANES 2021–2023). Points represent regression coefficients with 95% confidence intervals.

In the second approach, the “Treats and Diet Soda“ pattern was used as the reference. In the fully adjusted models, there was no evidence that the “Fast Food” pattern differed significantly from the “Treats and Diet Soda” pattern in their associations with BMI or waist circumference (Figure 3).

## 4 Discussion

In this nationally representative analysis of United States adults, three UPF dietary patterns were identified: “Fast Food,” “Treats and Diet Soda,” and “Lower UPF.” Both high‐UPF patterns were associated with significantly higher adiposity compared with the “Lower UPF” pattern, with no evidence of a difference between the two high‐UPF patterns. This study adds to the evidence by applying unsupervised clustering exclusively to UPF food groups, capturing how UPFs are consumed in combination and reflecting real‐world eating behaviors.

UPFs accounted for 54.3% of total energy intake in this sample, consistent with earlier NHANES estimates (56.1% in 2005–2014 [5]). As reported in previous studies [20, 21], UPF intake was disproportionately higher among socioeconomically deprived groups. Adjustment for sociodemographic and behavioral confounders attenuated the association between UPF intake and adiposity by approximately 26%–28%, indicating that part of the association reflects socioeconomic differences in UPF consumption, although a substantial proportion of this association remained. These findings are consistent with structural drivers of UPF consumption, including the lower cost of UPFs relative to minimally processed foods, targeted marketing in low‐income communities, and greater density of fast‐food and convenience outlets in disadvantaged areas [22, 23, 24], supporting the need for structural policy measures to reduce reliance on UPFs [2].

Associations between UPF intake and BMI and waist circumference remained significant after adjustment for energy intake and key dietary mediators, consistent with findings from observational studies across multiple populations [2, 4, 5, 6, 7, 8]. Dietary misreporting may contribute to observed associations in observational studies, as UPFs are often consumed rapidly and are more likely to be underestimated [10, 25]. However, associations persisted after adjustment using the ratio of reported energy intake to estimated energy requirements, indicating that misreporting alone is unlikely to explain the findings. These results support the hypothesis that the degree of processing itself may contribute to excess adiposity beyond calorie or nutrient content alone. Shared features of UPF‐rich diets may be relevant, including high energy density, rapid eating rate, soft texture, high palatability, altered food structure, and effects on satiety signaling, glycaemic responses, gut microbiota, and digestive efficiency [10, 26, 27].

The identified UPF patterns broadly map onto established dietary patterns in Western settings. Both the “Fast Food” and “Treats and Diet Soda” patterns resemble the “Western” pattern characterized by refined grains, processed meats, and sugary drinks, while “Lower UPF” aligns more closely with “Prudent” or “Healthy” patterns [14]. The two high‐UPF patterns differed in their dominant food sources: “Fast Food” was characterized by ready‐made fast foods, while “Treats and Diet Soda” included more low‐calorie or diet products and UPF ingredients that may be perceived as more health‐conscious alternatives.

Both high‐UPF dietary patterns were associated with higher BMI and waist circumference compared to the “Lower UPF” pattern, with no evidence of a significant difference between “Fast Food” and “Treats and Diet Soda” patterns when directly compared. These findings may suggest that overall UPF burden is more relevant for adiposity markers than specific UPF subtypes. However, they should be interpreted cautiously given the cross‐sectional design, potential residual confounding by broader dietary quality, socioeconomic conditions, food environment, and health behaviors not fully assessed in NHANES, and measurement error from two 24‐h recalls. Limited discriminatory power of UPF‐specific clustering may also have reduced the ability to detect differences between high‐UPF profiles. Reverse causation is possible, particularly for the “Treats and Diet Soda” pattern: individuals with higher adiposity may choose low‐calorie or diet UPF products as part of weight‐control efforts, making this pattern appear associated with adiposity when it may partly reflect a response to existing weight status.

Cohort studies of type 2 diabetes have reported lower risk for individual UPF items such as ultra‐processed bread, biscuits, breakfast cereals, and sweets [12, 13]. In this analysis, these items were consumed within broader high‐UPF patterns that remained associated with obesity indicators, highlighting the value of examining UPFs within dietary contexts rather than as isolated food items.

### 4.1 Strengths and Limitations

This study has several strengths. It was conducted in a large, nationally representative sample, enhancing generalizability to United States adults. UPF intake was estimated using two 24‐h dietary recalls with detailed disaggregation, improving the accuracy of Nova group estimates. Adjustment using the energy intake‐to‐expenditure ratio helped mitigate bias from dietary misreporting, which is particularly relevant for obesity‐related outcomes [25, 28]. A novel clustering approach based exclusively on UPF groups was applied, capturing consumption patterns rather than isolated food items and better reflecting real‐world eating behaviors.

Several limitations should be considered. The cross‐sectional design limits causal inference, as temporality between exposure and outcome cannot be established. Some misclassification of UPFs may have occurred due to limited information on food brands or preparation methods.

Dietary intake was self‐reported using two 24‐h recalls, which may not fully capture habitual intake and is vulnerable to recall bias and measurement error. Dietary misreporting may influence observed associations between eating patterns and adiposity, particularly in obesity‐related analyzes [25]. Although averaging two recalls and adjusting for the ratio of energy intake to estimated energy expenditure may reduce random error and some bias from energy misreporting, it cannot fully account for systematic underreporting or differential reporting across participant groups.

Selection bias due to non‐response and complete‐case restriction is possible. Sensitivity analyzes showed that excluded adults differed from the final analytic sample by age, income‐to‐poverty ratio, education, physical activity, and chronic disease prevalence. Adults missing BMI and/or waist circumference also differed from those with valid anthropometric measurements by age, income‐to‐poverty ratio, physical activity, smoking status, and chronic disease prevalence. These differences suggest that missingness was unlikely to be completely random. Because BMI and waist circumference were unavailable for participants with missing anthropometric data, the direction and magnitude of any resulting bias could not be assessed directly. Although NHANES survey weights account for differential selection, non‐response, and coverage, residual non‐response bias in anthropometric outcomes cannot be excluded.

Our clustering approach also has some important limitations. First, the clusters were derived exclusively from UPF food groups rather than from the whole diet. This was intentional, as our aim was to characterize heterogeneity within UPF consumption itself. However, this approach does not capture the broader dietary context in which UPFs are consumed, including foods that may accompany or offset these products within overall dietary patterns. It also naturally limits the contrast between clusters and may reduce between‐cluster variability. As a result, the identified clusters should be interpreted as UPF‐specific consumption profiles rather than as whole‐diet patterns. Nevertheless, the clusters still revealed meaningful associations with obesity indicators, suggesting that they capture relevant population‐level profiles of UPF consumption.

Finally, residual confounding may remain despite adjustment for a range of sociodemographic and behavioral covariates. In particular, NHANES did not allow full adjustment for total dietary quality, eating context, household food practices, food insecurity, or neighborhood food environment. This is important given the socioeconomic gradient observed in UPF consumption, meaning that associations with BMI and waist circumference may partly reflect broader social and environmental determinants of diet and obesity.

## 5 Conclusion

In this nationally representative sample of United States adults, higher UPF consumption was associated with greater BMI and waist circumference. UPF dietary patterns characterized by different dominant food sources showed broadly similar associations with adiposity. These findings from cross‐sectional data suggest that overall UPF intake may be an important marker of adiposity, but longitudinal studies are needed to assess temporality and causality. Further research should clarify whether these associations reflect processing itself, other features of UPF diets such as texture or energy density, or the broader dietary and socioeconomic context in which UPFs are consumed.

## Author Contributions

**Nadia Daniel:** conceptualization, methodology, formal analysis, writing – original draft, writing – review and editing, data curation, visualization, project administration. **Carmen Piernas:** writing – review and editing. **Paul Sherliker:** writing – review and editing, visualization. **Carolina Abreu de Carvalho:** conceptualization, methodology, formal analysis, investigation, writing – review and editing, writing – original draft, supervision. **Jennifer Carter:** conceptualization, methodology, supervision, writing – review and editing.

## Ethics Statement

NHANES protocols are approved annually by the National Center for Health Statistics Ethics Review Board. Written informed consent was obtained from all participants. The present secondary analysis of de‐identified public‐use data was exempt from additional institutional review.

## Conflicts of Interest

The authors declare no conflicts of interest.

## Transparency Statement

The corresponding author (Nadia Daniel) affirms that this manuscript is an honest, accurate, and transparent account of the study being reported; that no important aspects of the study have been omitted; and that any discrepancies from the study as planned have been explained.

## Supporting information


Supporting File

## Data Availability

The data that support the findings of this study are publicly available from the National Health and Nutrition Examination Survey website (CDC, 2025). These data were derived from publicly accessible resources and are available without restriction. Analytic code used for data processing, variable derivation, and statistical analyzes is openly available on the Open Science Framework at https://osf.io/r725z.
