# Supplementary material for: Ultra‐Processed Food Dietary Patterns and Obesity: A Cross‐Sectional Analysis of NHANES 2021–2023
Source: Health Sci Rep. 2026 Aug 19;9(8):e73062. doi: 10.1002/hsr2.73062 (PMC13487806; doi:10.1002/hsr2.73062)
Supplement: Supplementary file 1 [file HSR2-9-e73062-s001.docx]

## **Contents of supplementary material**

- **Supplementary Table S1.** Examples of foods included in each of the 33 food groups used in the cluster analysis
- **Supplementary Table S2.** Examples of the food groups excluded because they were consumed by less than 1% of the sample.
- **Supplementary Figure S2.** Average silhouette width across candidate clustering solutions in the k-means analysis of ultra-processed food consumption patterns.
- **Supplementary Table S3.** Mean food group intake (g or ml/day) by cluster membership in the 2-cluster solution, NHANES 2021-23
- **Supplementary Table S4.** Mean food group intake (g or ml/day) by cluster membership in the 4-cluster solution, NHANES 2021-23
- **Supplementary Table S5.** Mean food group intake (g or ml/day) by cluster membership in the 3-cluster solution, NHANES 2021-23
- **Supplementary Note S1.** Definition of chronic disease status
- **Supplementary Figure S3.** Flow diagram of participant selection for the analytic sample from NHANES 2021-2023.
- **Supplementary Table S6.** Comparison of adults included in and excluded from the final analytic sample.
- **Supplementary Table S7.** Comparison of adults with valid versus missing anthropometric measurements.
- **Supplementary Table S8.** Sociodemographic, lifestyle, and adiposity indicators (BMI and waist circumference) by cluster membership in the 3-cluster solution, NHANES 2021–2023.

**Supplementary Table S1.** Examples of foods included in each of the 33 food groups used in the cluster analysis

| **Food group** | **Examples of Items** |
| --- | --- |
| 1. Bread | White breads; Bagel, English muffin, garlic bread, brioche, French bread, cheese bread; pita bread, Cuban bread, etc. |
| 2. Breakfast cereal | Breakfast cereal from various brands; nutrition bar from various brands; granola. |
| 3. Cakes and biscuits | Different types of cakes and biscuits or cookies. |
| 4. Chips | Potato and other vegetable chips. |
| 5. Chocolates | Chocolate candy; dark or milk. |
| 6. Coated or breaded foods | Fast foods like chicken fried and coated; chicken tenders and nuggets; Onion rings; fish nuggets; seafood coated or breaded; hush puppy; bread crumbs; breading as ingredient. |
| 7. Coffee or tea | Different types of coffee or tea flavoured or sweetened; iced tea or coffee ready-to-drink; coffee creamer. |
| 8. Crackers | Crackers from various types and flavours (Butter, cheese, multigrain, saltine), rice cake crackers. |
| 9. Cream | Cream substitute powdered; heavy cream; flavoured cream; whipped cream. |
| 10. Desserts | Flan, puddings, syrups, pie crust, gelatine desserts, mousse, and sweetened canned fruits. |
| 11. Dips, gravies, other sauces | Ketchup, mayonnaise, mustard, barbecue sauce, salsa, gravies, etc. |
| 12. Doughnuts, sweet rolls, pastries | Doughnut various flavours, cinnamon bun, Danish pastry, chocolate croissant, fruit bread. |
| 13. Dressing | Salad dressings various flavours |
| 14. Fruit and sports drinks | Fruit-flavoured drinks, sports drinks, diet or sweetened enhanced water; flavoured water; cocktail juices; juices not made 100% from fruit. |
| 15. Ice cream | Ice cream various flavours; ice pops and frozen fruit juice bars. |
| 16. Low-calorie soft drinks | Low-calorie or sugar-free cola, fruit or flavoured soft drinks and energy drinks. |
| 17. Milk and chocolate drinks | Nutritional drink or shake; milk shake various flavours; nutritional powder mix; hot chocolate; chocolate drinks. |
| 18. Non-dairy milk | Almond, coconut, oat, rice and soy milks |
| 19. Pizzas and burgers | Pizzas various flavours; Hamburgers, cheeseburgers, chiliburgers from fast food |
| 20. Potato and dishes | French fries; mashed potato ready-to-eat; hash brown; potato scalloped from dry mix. |
| 21. Sandwiches | Hot dog, beef, chicken, pork and egg sandwiches; salami, turkey and ham sandwiches; peanut butter, almond butter, Nutella and jelly sandwiches. |
| 22. Sausages and dishes | Pork, beef or chicken sausages. |
| 23. Snacks | Corn snacks; tortilla chips; flavoured popcorn; pretzels; crackers sandwiches; trail mix with pretzels, cereal, or granola. |
| 24. Soft drinks | Cola, fruit or flavoured soft drinks; energy drinks |
| 25. Soup | Soup from dry, powder or read-to-serve various flavours |
| 26. Spirits and drinks | Distilled alcoholic beverages; martini; alcoholic cocktails; wine spritzer or cooler; liqueur; brandy; drinks (Moscow mule, Margarita, etc). |
| 27. Spreads | Sandwich spread; liver pate; cream cheese and imitation cheese; margarine. |
| 28. Sugar substitutes | Sucralose, saccharin, aspartame liquid or powder. |
| 29. Sweets | Candies; lollipop; fudge; chewing gum; marshmallow, etc. |
| 30. Tortillas | Corn, flour, whole wheat tortilla and taco shells. |
| 31. UPF fats | Industrial oils, oil cooking spray |
| 32. UPF meat | Salami, pepperoni, luncheon meat, turkey bacon, bologna, etc. |
| 33. Whole-grain bread | Whole wheat, whole grain, and multigrain breads. |

**Supplementary Table S2. Examples of the food groups excluded because they were consumed by less than 1% of the sample.**

| Food group | Examples of items |
| --- | --- |
| Beans, peas, legumes and dishes | Baked beans |
| Egg rolls and dumplings | Egg rolls beef, pork or meatless; Potsticker or wonton, potato dumplings. |
| Fish, seafood and dishes | Imitation crab and seafood restructured |
| Frozen meal | Frozen meals, ready-to-heat or vending machine preparations like: lasagna with meat, Salisbury steak with gravy, pasta with sauce, chicken and vegetable entree with noodles. |
| Other UPFs | Corn, yellow, from canned, cream style, Sunflower seeds, flavoured. |
| Pancakes and waffles | Industrialized pancakes and waffles plain or flavoured. |
| Pasta, noodles and dishes | Pasta or noodles from package mix, flavoured pasta. |
| Preparations bread-based | Bread and cornbread stuffing from dry mix. |
| Rice and dishes | Flavoured rice and rice from dry packet mix. |
| Smoothies and grain drinks | Industrialized fruit and vegetable smoothies; Horchata beverage; Whey protein beverages. |
| Soy and meat-alternative products | Vegetarian burger, bacon meatless, meatball meatless, chicken meatless. |
| Sweet or flavoured nuts | Nuts flavoured, sweetened or covered by chocolate. |
| Yoghurt | Yoghurts with flavours. |

**Supplementary Figure S1.** Elbow plot across candidate clustering solutions in the k-means analysis of ultra-processed food consumption patterns.

**Supplementary Figure S2.** Average silhouette width across candidate clustering solutions in the k-means analysis of ultra-processed food consumption patterns.

**Supplementary Table S3.** Mean food group intake (g or ml/day) by cluster membership in the 2-cluster solution, NHANES 2021-23

**Supplementary Table S4.** Mean food group intake (g or ml/day) by cluster membership in the 4-cluster solution, NHANES 2021-23

^1,2,3^Values with different superscript numbers differ significantly according to Tukey’s test (p<0.05).

**Supplementary Table S5.** Mean food group intake (g or ml/day) by cluster membership in the 3-cluster solution, NHANES 2021-23

| **Food groups** | **Cluster 1 – Fast Food (n=632)** | **Cluster 2 - Treats and Diet Soda (n=1039)** | **Cluster 3 – Lower UPF (n=2332)** | **p-value** |
| --- | --- | --- | --- | --- |
| Bread | 48.2^1^ | 99.1^2^ | 35.1^3^ | <0.001 |
| Breakfast cereal | 13.8^1^ | 22.4^2^ | 17.2^3^ | <0.001 |
| Cakes and biscuits | 48.9^1^ | 39.7^2^ | 35.0^2^ | <0.001 |
| Chips | 11.7^1^ | 15.1^2^ | 6.0^3^ | <0.001 |
| Chocolates | 6.1^1^ | 13.7^2^ | 5.5^1^ | <0.001 |
| Coated or breaded foods | 90.1^1^ | 18.1^2^ | 14.9^2^ | <0.001 |
| Coffee and tea | 108.0^1^ | 145.0^2^ | 53.3^3^ | <0.001 |
| Crackers | 6.0^1^ | 10.2^2^ | 6.1^1^ | <0.001 |
| Cream | 2.7 | 4.4 | 3.7 | 0.093 |
| Desserts | 13.9^1^ | 30.8^2^ | 12.0^1^ | <0.001 |
| Dips and gravy | 56.7^1^ | 41.4^2^ | 32.2^3^ | <0.001 |
| Doughnuts, sweet rolls and pastries | 17.7^1^ | 19.8^1^ | 7.8^2^ | <0.001 |
| Dressing | 15.1 | 15.4 | 15.7 | 0.647 |
| Fruit and sports drinks | 216.0^1^ | 210.0^1^ | 94.2^2^ | <0.001 |
| Ice cream | 34.3^1^ | 65.8^2^ | 18.6^3^ | <0.001 |
| Low-calorie soft drinks | 124.0^1^ | 280.0^2^ | 83.2^3^ | <0.001 |
| Milk and chocolate drinks | 49.5^1^ | 26.7^2^ | 27.8^2^ | <0.001 |
| Non-dairy milk | 8.9^1^ | 11.0^1^ | 29.2^2^ | <0.001 |
| Pizzas and burgers | 125.0^1^ | 42.7^2^ | 34.9^2^ | <0.001 |
| Potato and dishes | 110.0^1^ | 16.3^2^ | 9.4^3^ | <0.001 |
| Sandwiches | 29.0^1^ | 32.2^1^ | 9.4^2^ | <0.001 |
| Sausages and dishes | 17.7^1^ | 13.1^1^ | 7.8^2^ | <0.001 |
| Snacks | 23.6^1^ | 15.9^2^ | 11.8^3^ | <0.001 |
| Soft drinks | 439.0^1^ | 144.0^2^ | 77.1^3^ | <0.001 |
| Soups | 11.3 | 11.2 | 17.1 | 0.05 |
| Spirits and drinks | 41.4^1^ | 48.5^1^ | 25.3^2^ | <0.001 |
| Spread | 5.3^1^ | 7.7^2^ | 3.7^1^ | <0.001 |
| Sugar substitutes | 0.2^1^ | 0.5^2^ | 0.2^1^ | <0.001 |
| Sweets | 5.5^1^ | 7.4^2^ | 2.9^3^ | <0.001 |
| Tortillas | 19.9 | 19.2 | 18.6 | 0.779 |
| UPF fat | 1.8^1^ | 0.3^2^ | 0.2^2^ | <0.001 |
| UPF meat | 12.8^1^ | 53.4^2^ | 8.0^3^ | <0.001 |
| Whole grain bread | 2.9^1^ | 3.5^1^ | 8.5^2^ | <0.001 |

^1,2,3^Values with different superscript numbers differ significantly according to Tukey’s test (p<0.05).

**Supplementary Note S1.** Definition of chronic disease status

Chronic disease status was defined as self-reported physician diagnosis of at least one long-term condition, based on NHANES questionnaire data. Conditions included cardiovascular disease (heart failure, coronary heart disease, angina, myocardial infarction, or stroke), chronic respiratory disease (asthma or chronic obstructive pulmonary disease), thyroid disease, chronic liver disease (fatty liver disease, fibrosis, or cirrhosis), arthritis, or cancer. Participants reporting none of these conditions were classified as not having a chronic disease.

**Supplementary Figure S3.** Flow diagram of participant selection for the analytic sample from NHANES 2021-2023.

**Supplementary Table S6. Comparison of adults included in and excluded from the final analytic sample.** Values are unweighted n and survey-weighted percentage for categorical variables, or survey-weighted mean and standard error for continuous variables. P values were calculated using Rao-Scott adjusted chi-squared tests for categorical variables and survey-weighted linear regression for continuous variables. Percentages are calculated among participants with non-missing data for each characteristic.

| **Variable** | **Included analytic sample** | **Excluded adults (excluded due to missing dietary, anthropometric, or covariate data)** | **p values** |
| --- | --- | --- | --- |
| **Overall, n** | 4003 | 431 |  |
| **Sex, n (%)** |  |  | 0.206 |
| Male | 1761 (48.9) | 188 (44.8) |  |
| **Age (years)** | 48.7 (0.5) | 39.3 (1.8) | 0.0001 |
| **Race/Ethnicity** |  |  | 0.0687 |
| White | 2517 (62.0) | 235 (56.4) |  |
| Black | 446 (11.1) | 65 (13.7) |  |
| Hispanic/Other | 1040 (26.9) | 131 (29.8) |  |
| **Education level, n (%)** |  |  | 0.0498 |
| High school and below | 1205 (32.3) | 103 (40.8) |  |
| Some college | 1210 (29.7) | 90 (32.5) |  |
| College graduate | 1588 (38.1) | 88 (26.7) |  |
| **Income-to-poverty ratio** | 3.2 (0.1) | 2.6 (0.2) | 0.0009 |
| **Total moderate-vigorous activity (Continuous)** | 51.4 (2.6) | 67.7 (5.7) | 0.0315 |
| **Alcohol intake (drinks/day)** | 0.6 (0.03) | 0.4 (0.1) | 0.132 |
| **Smoking status, n (%)** |  |  | 0.6753 |
| Current smoker | 660 (17.6) | 78 (16.6) |  |
| **Chronic disease, n (%)** |  |  | 0.0168 |
| Yes | 2295 (48.2) | 205 (39.4) |  |

**Supplementary Table S7.** Comparison of adults with valid versus missing anthropometric measurements. Values are unweighted n and survey-weighted percentage for categorical variables, or survey-weighted mean and standard error for continuous variables. P values were calculated using Rao-Scott adjusted chi-squared tests for categorical variables and survey-weighted linear regression for continuous variables. Percentages are calculated among participants with non-missing data for each characteristic.

| **Variable** | **Adults with valid BMI and waist circumference** | **Adults missing BMI and/or waist circumference** | **p value** |
| --- | --- | --- | --- |
| **Overall, n** | 4259 | 175 |  |
| **Sex, n (%)** |  |  | 0.07 |
| Male | 1881 (48.9) | 68 (38.6) |  |
| **Age (years)** | 47.6 (0.6) | 56 (2.3) | 0.002 |
| **Race/Ethnicity** |  |  | 0.151 |
| White | 2651 (61.7) | 101 (55.2) |  |
| Black | 477 (11.1) | 34 (18.1) |  |
| Hispanic/Other | 1131 (27.2) | 40 (26.7) |  |
| **Education level, n (%)** |  |  | 0.076 |
| High school and below | 1235 (32.2) | 73 (45.9) |  |
| Some college | 1250 (30) | 50 (25.9) |  |
| College graduate | 1628 (37.7) | 48 (28.3) |  |
| **Income-to-poverty ratio** | 3.1 (0.1) | 2.7 (0.2) | 0.035 |
| **Total moderate-vigorous activity (Continuous)** | 53.3 (2.5) | 37.7 (5.9) | 0.028 |
| **Alcohol intake (drinks/day)** | 0.54 (0.03) | 0.49 (0.16) | 0.774 |
| **Smoking status, n (%)** |  |  | 0.019 |
| Current smoker | 711 (17.8) | 27 (11.1) |  |
| **Chronic disease, n (%)** |  |  | <0.001 |
| Yes | 2374 (46.7) | 126 (66.9) |  |

**Supplementary Table S8. Sociodemographic, lifestyle, and adiposity indicators (BMI and waist circumference) by cluster membership in the 3-cluster solution, NHANES 2021–2023.** Mean (standard error) presented unless otherwise indicated.

|  | Fast Food | Treats and Diet Soda | Lower UPF | p-value |
| --- | --- | --- | --- | --- |
| Overall | 632 (19.3) | 1039 (26.7) | 2332 (54.0) | <0.001 |
| Sex, n (%) |  |  |  |  |
| *Male* | 356 (62.6) | 528 (53.1) | 877 (41.9) | <0.001 |
| Age (years) | 43.0 (0.8) | 49.0 (0.7) | 50.6 (0.75) | <0.001 |
| Race and ethnicity (collapsed), n (%) |  |  |  |  |
| *White* | 389 (59.9) | 716 (69.6) | 1412 (59.0) |  |
| *Black* | 99 (16.4) | 102 (9.0) | 245 (10.3) |  |
| *Hispanic/Other* | 144 (23.6) | 221 (21.4) | 675 (30.7) | 0.006 |
| Education level (collapsed), n (%) |  |  |  |  |
| *High school and below* | 232 (40.2) | 281 (28.6) | 692 (31.2) |  |
| *Some college or AA degree* | 226 (35.1) | 334 (31.9) | 650 (26.7) |  |
| *College graduate* | 174 (24.7) | 424 (39.6) | 990 (42.1) | 0.003 |
| Income-to-poverty ratio | 2.8 (0.2) | 3.3 (0.1) | 3.2 (0.1) | 0.062 |
| Total moderate-vigorous activity (min/day) | 67.1 (6.3) | 48.2 (3.8) | 47.3 (2.5) | 0.014 |
| Drinks per day | 0.56 (0.1) | 0.6 (0.1) | 0.5 (0.0) | 0.277 |
| Smoking, n (%) |  |  |  |  |
| *Yes* | 160 (24.6) | 188 (19.5) | 312 (14.2) | 0.017 |
| Chronic diseases |  |  |  |  |
| *Yes* | 331 (41.3) | 604 (50.8) | 1360 (49.3) | 0.023 |
| Dietary variables |  |  |  |  |
| *UPF (%kcal)* | 66.0 (0.91) | 59.8 (0.79) | 46.1 (0.61) | <0.001 |
| *Total energy (kcal)* | 2452.2 (38.8) | 2352.7 (38.3) | 1772.0 (23.5) | <0.001 |
| *Saturated Fatty Acids (g)* | 33.2 (0.8) | 33.7 (0.9) | 23.2 (0.4) | <0.001 |
| *Polyunsaturated Fatty Acids (g)* | 24.7 (0.3) | 22.9 (0.6) | 17.3 (0.3) | <0.001 |
| *Sugars (g)* | 125.8 (4.1) | 113.7 (2.3) | 79.7 (1.2) | <0.001 |
| *Sodium (mg)* | 3863.2 (72.7) | 3749.3 (60.8) | 2769.7 (51.3) | <0.001 |
| *Fiber (g)* | 16.0 (0.4) | 17.0 (0.4) | 15.9 (0.5) | 0.081 |
| Body Mass Index (kg/m2) | 31.0 (0.4) | 30.5 (0.3) | 29.1 (0.3) | <0.001 |
| Waist circumference (cm) | 104.0 (1.1) | 103.3 (0.9) | 99.1 (0.7) | <0.001 |
